# Editorial: Epigenetic mechanisms and their involvement in rare diseases, volume II

**DOI:** 10.3389/fgene.2024.1483388

**Published:** 2024-09-20

**Authors:** Mojgan Rastegar

**Affiliations:** Department of Biochemistry and Medical Genetics, Max Rady College of Medicine, Rady Faculty of Health Sciences, University of Manitoba, Winnipeg, MB, Canada

**Keywords:** epigenetics, histone PTM, DNA methylation, chromatin, rare diseases, ribosomopathy

Epigenetic mechanisms are dynamic modes of gene regulation that are continually in place during life. These regulatory mechanisms control gene expression and cellular phenotypes without being directly reflected by the genomic DNA sequences that carry the genetically coded information. Diverse modes of epigenetic mechanisms exist; among them, epigenetic modifications are perhaps the most well-studied. The best-known epigenetic modifications are histone post-translational modifications (PTM) and chemical alteration of DNA molecules, known as DNA methylation. As research progresses, the impacts of epigenetic mechanisms have become increasingly evident in various human diseases, including rare diseases. Volume I of this special topic focused on a diverse range of original research and review articles (Rastegar and Yasui, 2021) that covered the role of epigenetics in a variety of rare diseases.

This current Research Topic includes articles on the epigenetic basis of rare neurological diseases authored by Moss et al. and Roberts et al., as well as the impact of epigenetics in other rare diseases covered by Fu et al. and Lyu et al.

In a comprehensive review, Moss et al. discuss the UBTF E210K neuroregression syndrome as part of ribosomopathy diseases. The authors focus on UBF, which is an epigenetic regulator of ribosomal DNA (*rDNA*) genes and a transcriptional regulator acting at the level of chromatin structure. Indeed, UBF controls the open chromatin state at the *rDNA* genes and a basal *trans*-acting factor directing *rRNA* transcription by RNA polymerase I. Through a series of interesting concepts on *rRNA* transcription, the authors cover a large volume of current literature on *47S primary rRNA* transcription and processing into smaller mature *rRNA* molecules. They comprehensively discuss UBF as a multi-HMGB-box protein and the role of different variants in human disease. This article is further complemented with five illustrations that are elegantly designed to capture the main points of this manuscript (Moss et al.).

In a recent article, Roberts et al. review the main epigenetic mechanisms involved in rare neurological diseases. This manuscript begins with the definition of rare diseases and an illustration of the prevalence of a disease that is considered a “rare disease” based on its geographic location. The authors provide a comprehensive and up-to-date overview of the etiology, clinical symptoms, associated epigenetic basis, and genetic causes of selected neurological diseases. This includes the following diseases: Rett syndrome, Rubinstein–Taybi syndrome, Angelman syndrome, Prader–Willi syndrome, and Huntington’s disease. The article includes six figures and a table. As an example of a rare disease, Rett syndrome, which is caused by the *MECP2* mutation, the authors describe the functional role of MeCP2 protein and its isoforms, different developmental stages of the disease, *MECP2* gene structure, the impact of its genetic mutations on the functional role of MeCP2 as a DNA methylation-binding protein, its regulatory role on RNA splicing and the global epigenomics of brain cells. Additional discussions on MeCP2 protein function include regulation of the chromatin structure, ribosomal targets, and potential impact on other epigenetic modifications (Roberts et al.).

Fu et al. focus on rare diseases of epigenetic origin (RDEOs). The authors present a global perspective and discuss the application of next-generation sequencing and bioinformatics in the associated pathophysiology in the affected patients. Their comprehensively prepared Table 1 highlights the role of DNA methylation, histone PTM, and other modes of epigenetic regulations, such as chromatin remodeling, in association with epigenetic factors, the causative genetic basis of specific RDEOs. The OMIM entry is also presented for each of these rare diseases of epigenetic origin. The authors further discuss the challenges and potential opportunities in the study of rare diseases of epigenetic origin. This article is complemented by two well-designed illustrations (Fu et al.).

In another article, Lyu et al. provide an overview of the role of DNA modifications in liver fibrosis. The authors discuss the pathogenesis of hepatic fibrosis and describe liver fibrosis as a repair mechanism for the damage and injury of the liver. They then present evidence from published literature to link this process to DNA methylation. The article covers the basis of hepatic stellate cell activation in the case of liver damage and/or inflammation. Specific factors and cell signaling pathways that play key roles in this process are discussed. This article presents an overview of the application of nucleoside and non-nucleoside analogs in terms of their use in inhibiting DNA methylation. Discussed inhibitors of DNA methylation from the nucleoside group include 5-azacytidine and 5-aza-deoxycytidine, zebularine, and guadecitabine. The authors also discuss non-nucleoside analogs that are inhibitors of DNA methylation. This article is complemented with a figure that shows the connection between liver injury, activation of hepatic stellate cells, DNA methylation, and gene regulation (Lyu et al.).
